# Supplementary material for: GO/Bi2S3 Doped PVDF/TPU Nanofiber Membrane with Enhanced Photothermal Performance
Source: Int J Mol Sci. 2020 Jun 13;21(12):4224. doi: 10.3390/ijms21124224 (PMC7352882; doi:10.3390/ijms21124224)
Supplement: Supplementary file 1 [file ijms-21-04224-s001.pdf]

Supplementary Materials

GO/Bi_2S_3 Doped PVDF/TPU Nanofiber Membrane with Enhanced Photothermal Performance

Wenxiu Yang [1,†], Yonggui Li [2,†], Long Feng [1], Yimiao Hou [3,4], Shuo Wang [1], Bo Yang [1] and Xuemin Hu [1,*], Wei Zhang [1] and Seeram Ramakrishna [5,*]

1 College of Textile and Garments, Hebei Province Textile and Garment Technology Innovation Center, Hebei University of Science and Technology, Shijiazhuang 050018, China; wenxiuyang-hbust@outlook.com (W.Y.); fenglongxxl@outlook.com (L.F.); fzwangshuo@hebust.edu.cn (S.W.); yangbo564@outlook.com (B.Y.); bobzhang69@outlook.com (W.Z.)

2 Fujian Key Laboratory of Novel Functional Textile Fibers and Materials, Minjiang University, Fuzhou, Fujian 350108, China; LiYonggui@mju.edu.cn

3 School of Environmental Science and Technology, Hebei University of Science and Technology, Shijiazhuang 050018, China; ws15222329526@outlook.com

4 National Joint Local Engineering Research Center for Volatile Organic Compounds and Odorous Pollution Control, Shijiazhuang 050018, China;

5 Center for Nanofibers & Nanotechnology, Nanoscience & Nanotechnology Initiative, Faculty of Engineering, National University of Singapore, Singapore 117576, Singapore;

* Correspondence: huxuemin@hebust.edu.cn (X.H.); seeram@nus.edu.sg (S.R.); Tel.: +86-311-81668835 (X.H.)

† These authors contributed equally to this work.

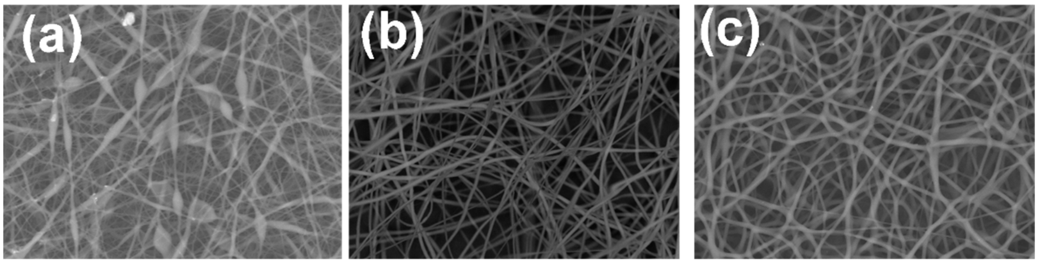

Figure 1S. The SEM images of PVDF with different concentrations of (**a**) 10%, (**b**) 11% and (**c**) 12%.

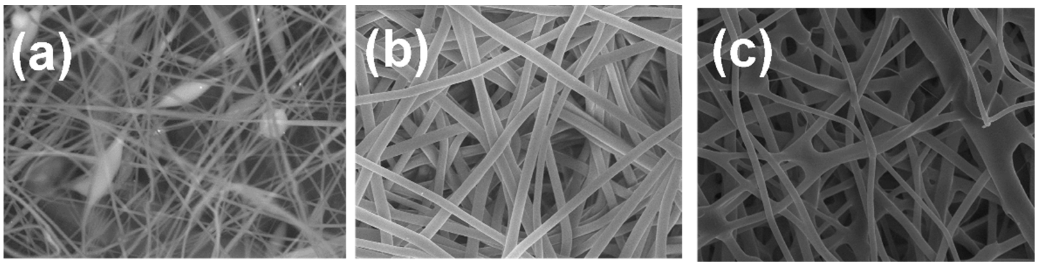

Figure 2S. The SEM images of TPU with different concentrations of (**a**) 23%, (**b**) 24% and (**c**) 25%.

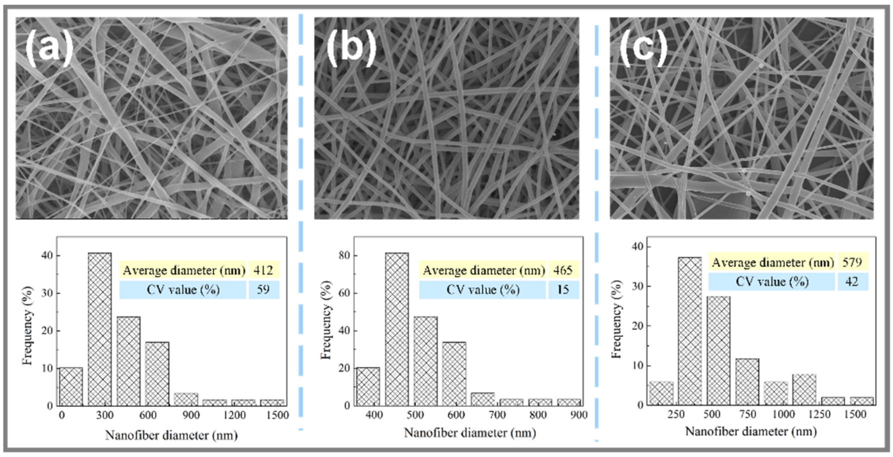

Figure 3S. The SEM images of PVDF/TPU with different solution mass ratio of (**a**) 10%, (**b**) 11% and (**c**) 12%. Below each SEM image is the corresponding diameter distribution, average fiber diameter and CV value.

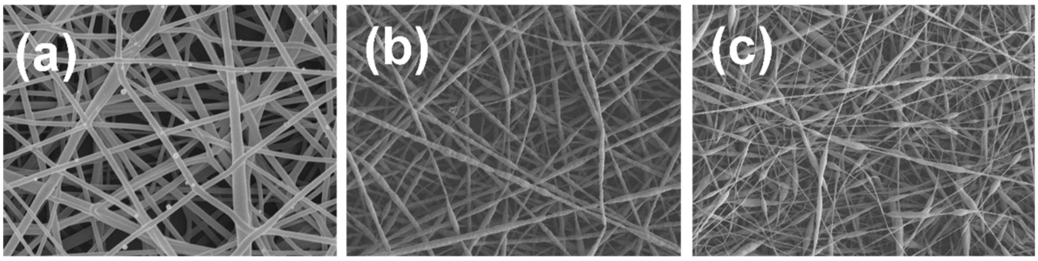

Figure 4S. The SEM images of GO-PVDF/TPU with different GO NPs concentrations of (**a**) 10%, (**b**) 11% and (**c**) 12%.

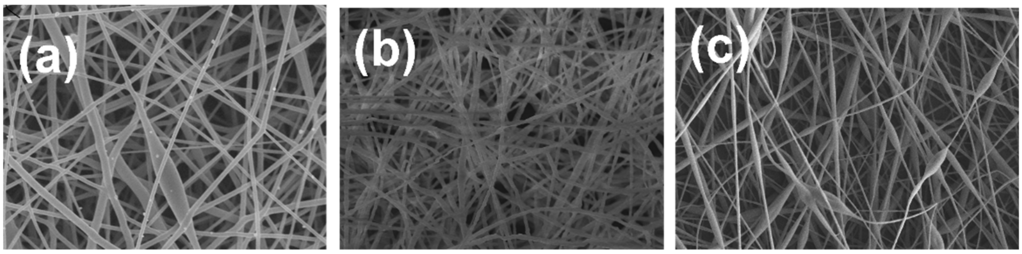

Figure 5S. The SEM images of GO/Bi_2S_3-PVDF/TPU with different GO/Bi_2S_3 NPs concentrations of (**a**) 10%, (**b**) 11% and (**c**) 12%.

Table S1. Mechanical property of different membranes.

	PVDF	TPU	PVDF/TPU	GO-PVDF/TPU	GO/Bi2S3-PVDF/TPU
Maximum strength (MPa)	15.3	19.7	17.6	20.1	20.4
Elongation at Break (%)	55	132	113	122	119
